# Supplementary material for: Population Pharmacokinetic Model of Adalimumab Based on Prior Information Using Real World Data
Source: Biomedicines. 2023 Oct 18;11(10):2822. doi: 10.3390/biomedicines11102822 (PMC10604709; doi:10.3390/biomedicines11102822)
Supplement: Supplementary file 1 [file biomedicines-11-02822-s001.zip › biomedicines-2542597-supplementary.pdf]

Supplementary material

Figure S1. Structural model and Monolix code of the final model.
Figure S2. Goodness of fit plot for the reference and the final model.
Figure S3. NPDE of the reference and the final model.

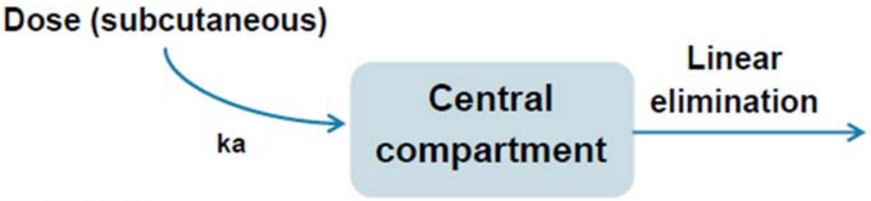

DESCRIPTION:
The administration is extravascular with a first order absorption (rate constant ka). The PK model has one compartment (volume V) and a linear elimination (clearance Cl).

```
[LONGITUDINAL]
input = {ka, V, Cl, beta1, AAA, beta2, ALB}
AAA={use=regressor}
ALB={use=regressor}

EQUATION:
if AAA==0
            ClwithAAA=Cl
elseif AAA==1
            ClwithAAA= Cl * (1+ beta1)
end
            ClwithALB= (ALB/3.77)^beta2

PK:
; PK model definition
Cc = pkmodel(ka, V, Cl=ClwithAAA*ClwithALB)

OUTPUT:
output = Cc
```

Figure S1. Structural model and Monolix code of the final model.

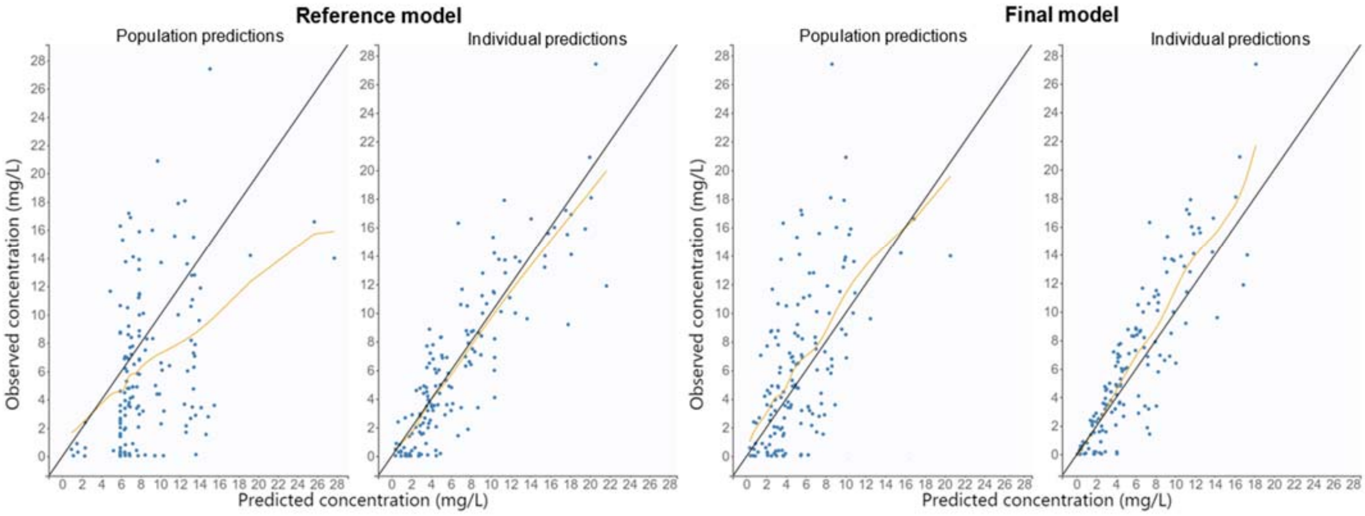

Figure S2. Goodness of fit plot for the reference and the final model.

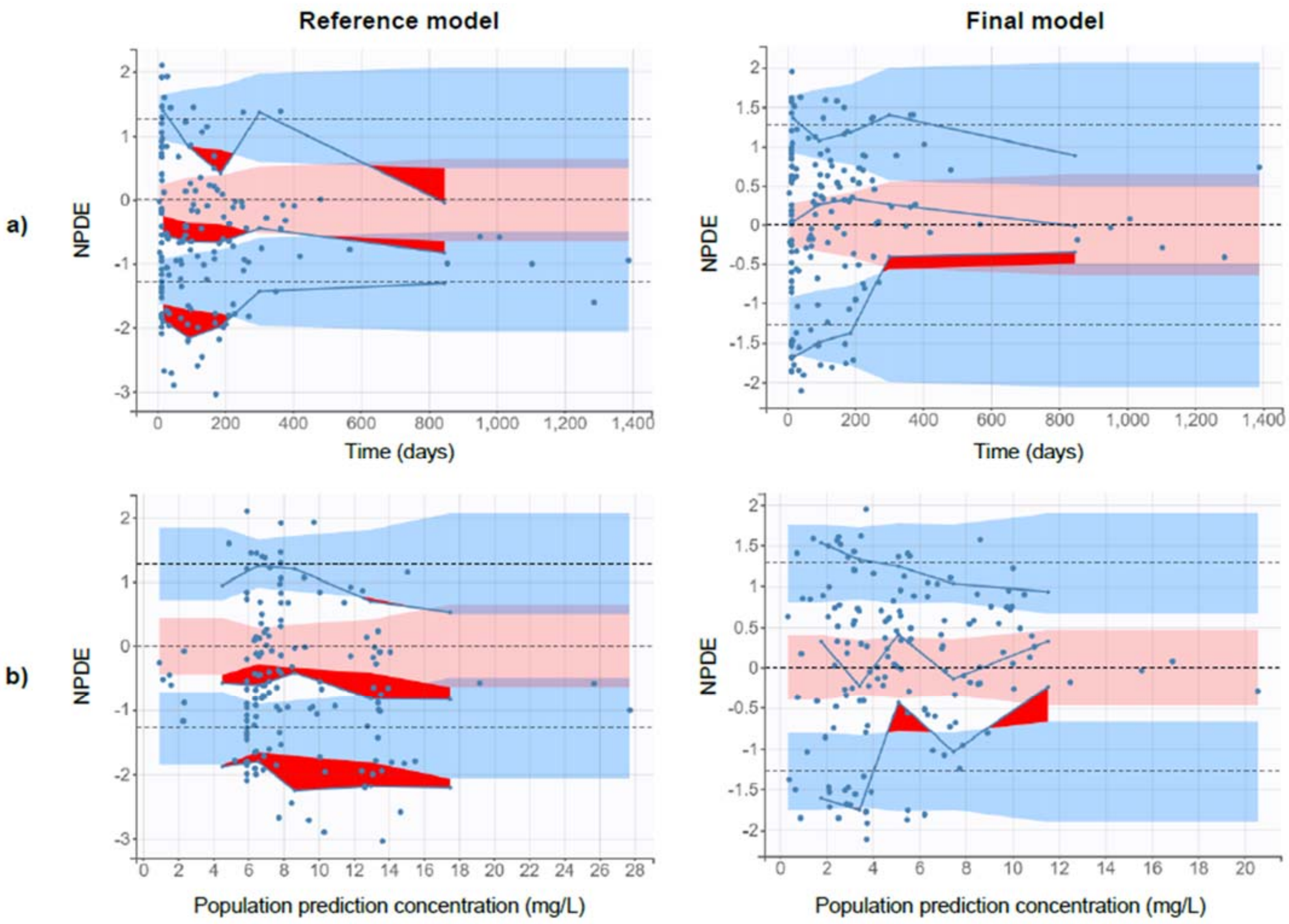

Figure S3. NPDE of the reference and the final model. (a) Plot of NPDE versus time. (b) Plot of NPDE versus population predicted concentration. Blue solid lines are the lines corresponding to 0, 5% and 95% critical values; black dashed lines, prediction intervals; blue-shaded area, 90% confidence interval (CI) of the 5% and 95% critical values; pink-shaded area, 90% CI of 0; red-shaded area, outliers of the bounds of the CI.

Disclaimer/Publisher's Note: The statements, opinions and data contained in all publications are solely those of the individual author(s) and contributor(s) and not of MDPI and/or the editor(s). MDPI and/or the editor(s) disclaim responsibility for any injury to people or property resulting from any ideas, methods, instructions or products referred to in the content.
